# Thymosin β-4 is a novel regulator for primary cilium formation by nephronophthisis 3 in HeLa human cervical cancer cells

**DOI:** 10.1038/s41598-019-43235-1

**Published:** 2019-05-02

**Authors:** Jae-Wook Lee, Hong Sug Kim, Eun-Yi Moon

**Affiliations:** 1 Department of Bioscience and Biotechnology, Sejong University, Seoul, 05006 Republic of Korea; 2 Macrogen Inc., 254, Beotkkot-ro, Geumcheon-gu, Seoul, 08511 Republic of Korea

**Keywords:** Cancer, Cell biology

## Abstract

Thymosinβ-4(Tβ4) is an actin-sequestering protein involved in tumor malignancy. Primary cilia, microtubule-based organelles, are present in most eukaryotic cells, which might be related to tumor cell transformation. Here, we investigated whether ciliogenesis is affected by Tβ4 in HeLa human cervical cancer cells. The inhibition of Tβ4 attenuated primary cilia formation. The frequency of cilia was increased by Tβ4 overexpression. When yeast two-hybrid assay was performed by using Tβ4 as a bait, we rescued nephronophthisis 3(NPHP3), one of the components of primary cilia. Interaction of Tβ4 with NPHP3 in mammalian cells was confirmed by GST-pulldown assay. Their intracellular co-localization was observed by immunofluorescence staining at peripheral surface of cells. In addition, the number of ciliated cells was reduced by the inhibition of NPHP3. Moreover, NPHP3 expression was decreased by the inhibition of Tβ4 but it was increased by Tβ4 overexpression. Taken together, the results demonstrate that primary cilia formation could be regulated by Tβ4 through its interaction with NPHP3 and/or the control of NPHP3 expression. It suggests that Tβ4 is a novel regulator for primary cilia formation by NPHP3. It also suggests that tumorigenesis could be associated with inappropriate regulation of Tβ4 and/or NPHP3 expression to maintain primary cilia formation normally.

## Introduction

Primary cilia are microtubule-based and antenna-like organelles protruded out of plasma membrane surface in most vertebrate cells. Since primary cilia transduced diverse intracellular signaling involved in embryonic development and tissue homeostasis, the abnormality of primary cilia is major causes of development disorders and human diseases such as cancer^1–3^. Defect of primary cilia frequency and length has been found in many types of pre-malignant and invasive tumor cells^4–6^. Several antitumor chemicals inhibit the proliferation of human pancreatic ductal cancer cells by restoring primary cilia formation^7^. In contrast, relatively high frequency of primary cilia has been found in some highly proliferative human cancer cells such as HeLa cervical carcinoma and MG63 osteosarcoma^8^. Besides, primary cilia are required for smoothened-driven tumor growth^9,10^. In addition, the disruption of primary cilia by hedgehog pathway inhibitor-4 abolishes malignant properties of chondrosarcoma cells^11^. Although many researches to correlate primary cilia with tumorigenesis have been reported, the effect of ciliogenesis in tumor cells remains contradictable and elusive. Therefore, the study of ciliogenesis in tumor cells would reveal pathological mechanisms leading to primary cilium-associated tumorigenesis and malignant tumors.

Thymosin β-4 (Tβ4), natural occurring 43-amino acid small peptide, is ubiquitously expressed in any type of cells and all tissues except red blood cells^12–14^. Tβ4 sequesters globular actin (G-actin) monomer to regulate actin cytoskeleton dynamics^15^. Tβ4 overexpression is highly associated with tumor malignancy in many types of cancers^16,17^. Tβ4 increases the resistance to paclitaxel-induced apoptosis by the inhibition of caspase-3 activation^18^, ERK activation^19^, and reactive oxygen species (ROS)-mediated HIF-1α stabilization^20^. In addition, Tβ4 increases metastatic properties such as cell migration, and angiogenesis in many types of cancer cells through various mechanisms including the regulation of actin cytoskeletal reorganization^21^, the induction of HIF-1α and VEGF^16,22^, the regulation of GSK-3 activity^23^, and the activation of Rac1-GTPase and Rap1-GTPase^17,24^. In present study, we rescued a ciliary protein, nephronophthisis3 (NPHP3), in yeast two hybrid screening by using Tβ4 as a bait.

NPHP3 is ciliary protein localized in basal body and centrioles of primary cilia. Mutations of *NPHP3* are responsible for adolescent nephronophthisis (NPHP) which is autosomal recessive poly cystic kidney disorder and the most frequent genetic disease of the renal failure in children and young adults^25–27^. NPHP is considered as one of the ciliopathies caused by ciliary dysfunction^28^. Homomorphic mutation of *nphp3* allele turns out to be the defect of primary cilia length control in epithelial mouse kidney cells^29^. Knockdown of zebrafish ortholog *nphp3* with morpholino oligo reduces the frequency and the length of primary cilia in Kupffer’s vesicle^30^.

Here, we investigated whether Tβ4 regulates ciliogenesis and whether Tβ4 and NPHP3 cooperate in primary cilia formation in HeLa cervical cancer cells. Our data showed that Tβ4 was interacted with NPHP3 at the cortical cell surface. Our data also showed that primary cilia formation was inhibited by the inhibition of Tβ4 or NPHP3 expression. In addition, NPHP3 expression was dependent on the alteration of Tβ4 expression. It suggests that Tβ4 could be associated with the localization and the expression of NPHP3, which modulates the formation of primary cilia in tumor cells.

## Results

### Primary cilia formation was regulated by the alteration of Tβ4 expression

**Figure 1:**
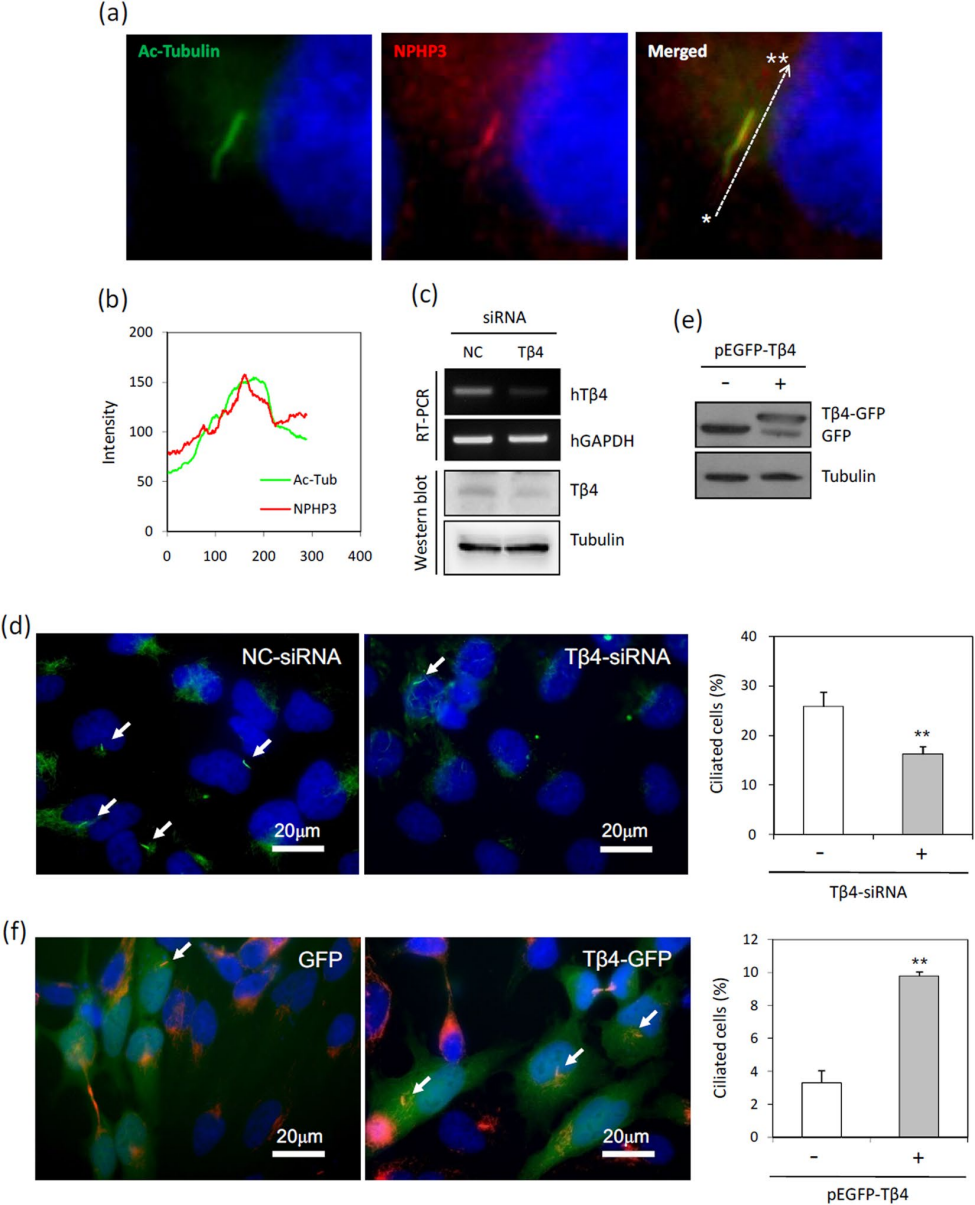
Effect of Tβ4 on primary cilia formation in HeLa cells. (**a**) HeLa ells were incubated in serum-starved media with 0.1% FBS for 36 h. The cells were fixed and stained with antibody against Ac-tubulin (green) or NPHP3 (red). The representative fluorescence image of primary cilia was shown. (**b**) Overlay of fluorescence intensity of Ac-tubulin (green) and NPHP3 (red) through the whole length of primary cilia was shown in line graph (Line scan * → ** in **a, right**). (**c**,**d**) Cells were transfected with AccuTarget™ negative control siRNA (NC) or Tβ4-siRNA for 24 h. (**c**) The mRNA (upper) and protein (lower) expression of Tβ4 were shown. (**d**) The cells were incubated in serum-starved media for 36 h, fixed and stained with antibody against Ac-tubulin (green) and DAPI (blue). The ciliated cells in AccuTarget™ negative control siRNA-treated (white) and Tβ4-knockdown cells (grey) were counted (n > 500 cells). (**e,f**) Cells were transfected with pEGFP-2B or pEGFP-Tβ4 plasmid for 24 h. (**e**) The expression of GFP and Tβ4-GFP were detected with GFP antibody. (**f**) The cells were fixed and stained with antibody against Ac-tubulin (red) and DAPI (blue). The ciliated cells in GFP (white) or Tβ4-GFP-positive cells (grey) were counted. Processing (such as changing brightness and contrast) is applied equally to controls across the entire image. Data in a bar graph represent the means ± SEM. **p < 0.01; significantly different from control cells.

Even though it is difficult to detect primary cilia in many types of cancer cells^4,5^, it has been reported that relatively high frequency of primary cilia were observed by using serum-starved culture condition in HeLa cervical cancer cells^8^. In addition, many researchers reported that primary cilia formation was induced by the incubation with low percentage of serum^31–34^. Our data also showed that high percentage of HeLa cells significantly expressed primary cilia (24.6 ± 0.39%) under serum-starved condition (Supplementary Fig. S1). Primary cilia were visualized by immunofluorescence staining to acetylated (Ac-) tubulin, a fundamental component of primary cilia structure, and NPHP3, a ciliary protein (Fig. 1a). The fluorescence by Ac-tubulin was overlapped with NPHP3 along the almost entire length of cilium (Fig. 1b).

We examined the effect of Tβ4 on primary cilia formation. Tβ4 expression was inhibited by small interfering RNA, mRNA and protein expression levelof Tβ4 was reduced (Fig. 1c). The frequency of primary cilia was significantly decreased about 37.3% in Tβ4-knockdown cells as compared to that in control cells under serum starvation (Fig. 1d). In addition, we studied whether Tβ4 expression affects primary cilia formation in the presence of serum. HeLa cells were transfected with pEGFP-C2 control plasmid DNA or pEGFP-Tβ4 plasmid DNA. Expression of GFP and Tβ4-GFP was detected by western blot (Fig. 1e). The frequency of primary cilia dramatically increased in Tβ4-GFP-positive cells (9.8 ± 0.26%) as compared to that in GFP-positive cells (3.3 ± 0.73%) (Fig. 1f). These results suggest that primary cilia formation could be regulated by Tβ4 expression in HeLa cervical cancer cells.

### Tβ4 and NPHP3 were interacted and co-localized at the peripheral surface of cells

**Figure 2:**
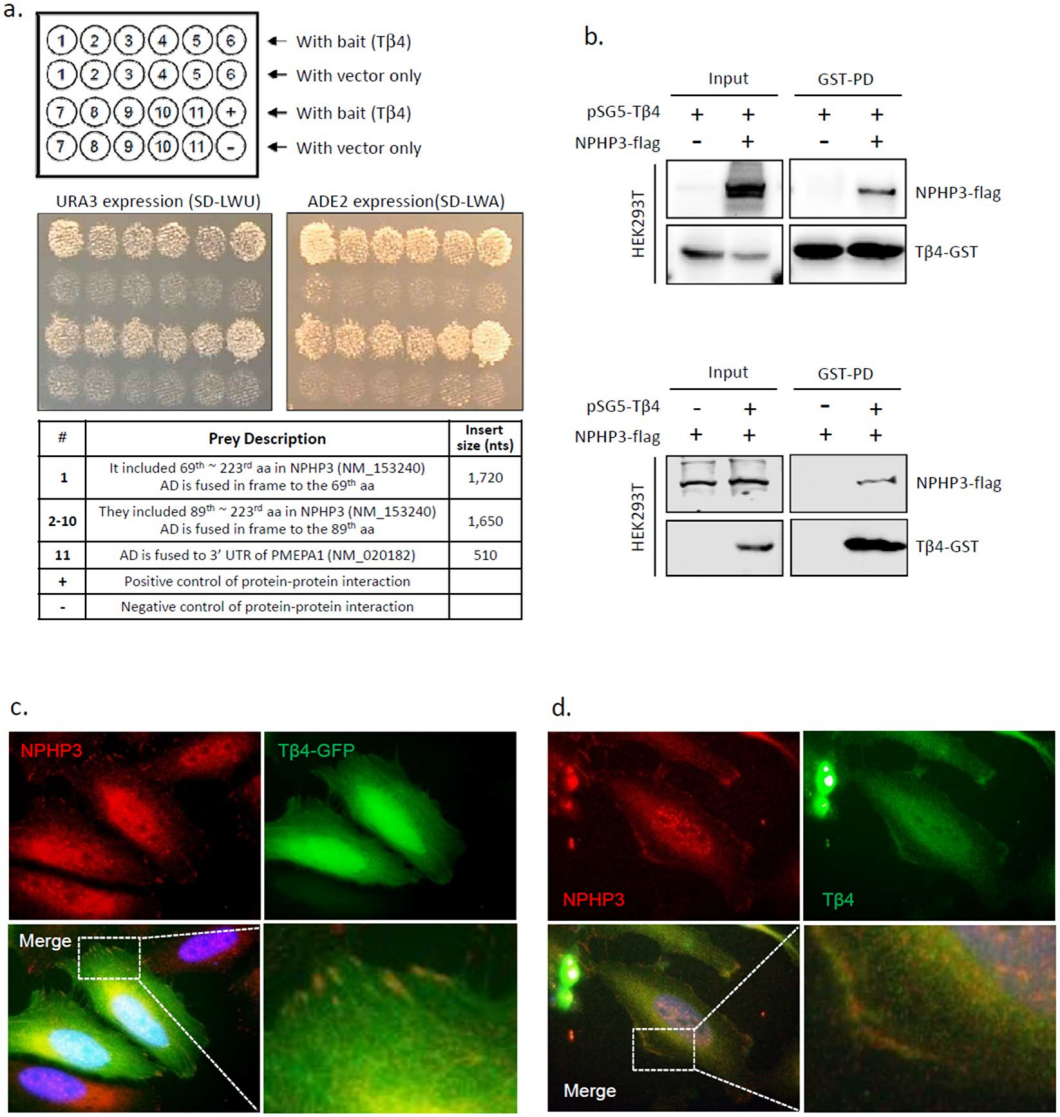
Interaction of Tβ4 and NPHP3 by Y2H, GST-pulldown and immunofluorescence staining. (**a**) The yeast two-hybrid assay with Tβ4 was performed in yeast PBN204 strain containing reporters that are under control of different GAL4-AD fusion proteins. Baits were expressed as GAL4 DNA-BD fusion Tβ4 in the pGBKT plasmid; preys were expressed as GAL4-AD fusion proteins in pACT2 vector. Yeast transformants of Tβ4 bait and GAL4-AD fusion proteins were spread on SD-LWU and SD-LWA. The growth of 1~11 yeast colonies on two selection medium indicated the interaction between Tβ4 and NPHP3. Insert size presented how many nucleotides (nts) are in between *EcoRI* and *XhoI* for each prey. (**b**) HEK 293 T cells were transfected with pSG5-Tβ4 plasmid with or without pCDNA6-NPHP3 plasmid (top). HEK 293 T cells were transfected with pCDNA6-NPHP3 plasmid with or without pSG5-Tβ4 plasmid (bottom). NPHP3-flag binding Tβ4 was rescued by GST-pulldown assay using glutathione agarose 4B bead. NPHP3-flag or Tβ4-GST was detected with antibodies against flag or GST, respectively. (**c**) HeLa cells were transfected with pEGFP-Tβ4 plasmid for 24 h. And then cells were fixed and stained with antibodies against NPHP3. (**d**) HeLa cells were stained with antibodies against endogenous Tβ4 and NPHP3. Tβ4 was visualized with Alexa 488-conjugated secondary antibody. (**c,d**) NPHP3 was visualized with Alexa 568-conjugated secondary antibody. Nucleus was stained with DAPI. The representative fluorescence image of Tβ4 (green), NPHP3 (red), and merged color (lower left) were shown. Dotted rectangle indicates co-localization of Tβ4 and NPHP3 at peripheral cell surface. The dotted rectangle in merged image (lower right) was magnified 4000×. Processing (such as changing brightness and contrast) is applied equally to controls across the entire image.

To study the interaction of Tβ4 with other proteins to control primary cilia formation, we performed yeast two-hybrid (Y2H) screening assay using Tβ4 bait, hybrid library of the human thymus cDNA activation domain (AD) and yeast PBN204 strain with three reporters, *URA3*, *ADE2*, and *lacZ*. 120 candidate preys were initially selected, which satisfied three reporter gene expressions. To reduce the possibility of picking up false positive candidates through the activation of reporter gene expression by AD fusion protein, we confirmed the interaction of each prey with Tβ4 bait by re-introducing each prey’s DNA and Tβ4 bait (Fig. 2a, top). Then, 11 positive colonies were selected (Fig. 2a, middle). Insert size of prey #1, #2~#10 and #11 was 1,720, 1,650 and 510 nucleotides (nts), respectively. Each prey was identified by nucleotide alignment in NCBI blast following DNA sequencing (prey #1~#10 and #11) (Supplementary Fig. S2) or restriction enzyme digestion (prey #8~#10). Forward or reverse insertion site was confirmed by the existence of *EcoRI* or *XhoI* sequence, respectively. AD was exactly fused to each prey’s DNA, which indicated its interaction with DNA binding domain of GAL4 (GAL4-BD) in Tβ4 bait. Prey #1 included 69^th^~223^rd^ aa in NPHP3 (1,330 aa in total). AD in prey #1 was fused to 259^th^ nucleotide, cytosine in CTG that encodes the 69^th^ amino acid (aa) of NPHP3. Prey #2~#10 included 89^th^~223^rd^ aa in NPHP3. AD in prey #2~#7 was fused to 319^th^ nucleotide, guanine in GCC that encodes the 89^th^ aa of NPHP3. AD in prey #8~#10 was fused to NPHP3 at the same site in prey #2~#7. As a whole, preys #2~#10 are all same genes that encode NPHP3. AD in prey #11 is fused to 1,272^nd^ nucleotide, cytosine of 3′ UTR of prostate transmembrane protein, androgen induced 1 (PMEPA1) transcript variant 1 (NM_020182; cds 394-1,257; 997 aa in total). In addition, 3′ UTR sequence of PMEPA1 was translated into peptide sequence and aligned it to find any gene in NCBI blast. However, no information was found for 3′UTR colony #11. Therefore, these results demonstrate that 10 real positive colonies (prey #1~#10) encode NPHP3 (NM_153240) except one colony (prey #11) encoding 3′ UTR sequence of PMEPA1 mRNA (Fig. 2a, bottom).

Based on the findings in yeast two-hybrid screening assay using DNA library established from parts of full length of each gene, we tested the direct interaction between Tβ4 and NPHP3 in mammalian cells using glutathione-S-transferase (GST)-pulldown assay in HEK293T cells (Fig. 2b). No interaction was detected in the presence of Tβ4 bait (Fig. 2b, top) or NPHP3 prey (Fig. 2b, bottom) alone. To examine the distribution compartment of Tβ4 and NPHP3 throughout cells, HeLa cells were transfected with pEGFP-Tβ4 and immunostained with antibodies against NPHP3. Immunofluorescence data showed that endogenous NPHP3 was dot-like structure especially at the cell periphery in non-ciliated cells. In addition, NPHP3 was co-localized with Tβ4 at the cortical cell surface (Fig. 2c). This implicated that NPHP3 could interact with Tβ4 at the peripheral surface of cells. The result was confirmed by the interaction of endogenous Tβ4 with endogenous NPHP3 (Fig. 2d). Although both proteins are expressed in many intracellular compartments, we focused on their co-localization at the cortical cell surface with stronger signal than other compartment. These consequences led us to hypothesize that Tβ4 may interact with NPHP3 around apical cell surface where effectively serve NPHP3 into ciliary compartment. Then, it suggests that it might be the relation between this apical cell surface co-localization and the presence of NPHP3 into ciliary compartment.

### Tβ4 regulated NPHP3 expression which is necessary for primary cilia formation

**Figure 3:**
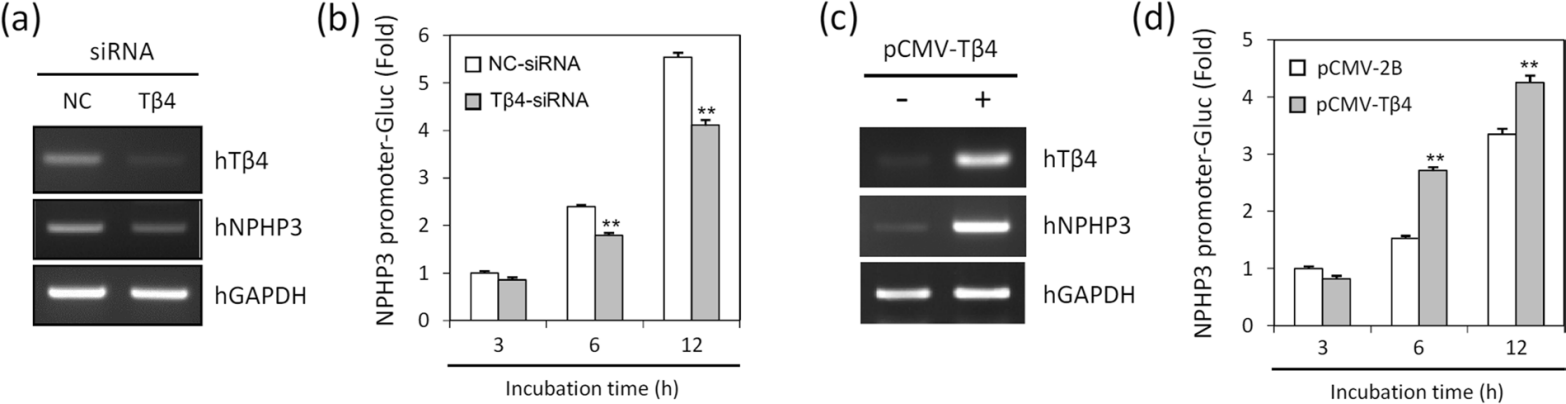
Effect of Tβ4 on NPHP3 expression in HeLa cells. (**a,b**) HeLa cells were transfected with AccuTarget™ negative control siRNA (NC) or Tβ4-siRNA for 24 h. (**a**) Expression level of Tβ4 and NPHP3 transcripts were measured by RT-PCR. (**b**) HeLa cells were co-transfected with pEZX-PG02-NPHP3-promoter Gaussia luciferase (Gluc) plasmid. The activity of Gluc in cultured media was measured with luminometer using Gluc substrate. Bar graph represents the mean of NPHP3-promoter activity. (**c,d**) HeLa cells were transfected with pCMV-2B or pCMV-Tβ4 for 24 h. (**c**) Expression level of Tβ4 and NPHP3 transcripts were measured by RT-PCR. (**d**) HeLa cells were co-transfected with pEZX-PG02-NPHP3-promoter Gluc plasmid. Gluc activity in cultured media was measured with luminometer using Gluc substrate. Bar graph represents the mean of NPHP3-promoter activity. Processing (such as changing brightness and contrast) is applied equally to controls across the entire image. Data in a bar graph represent the means ± SEM. **p < 0.01; significantly different from control cells.

**Figure 4:**
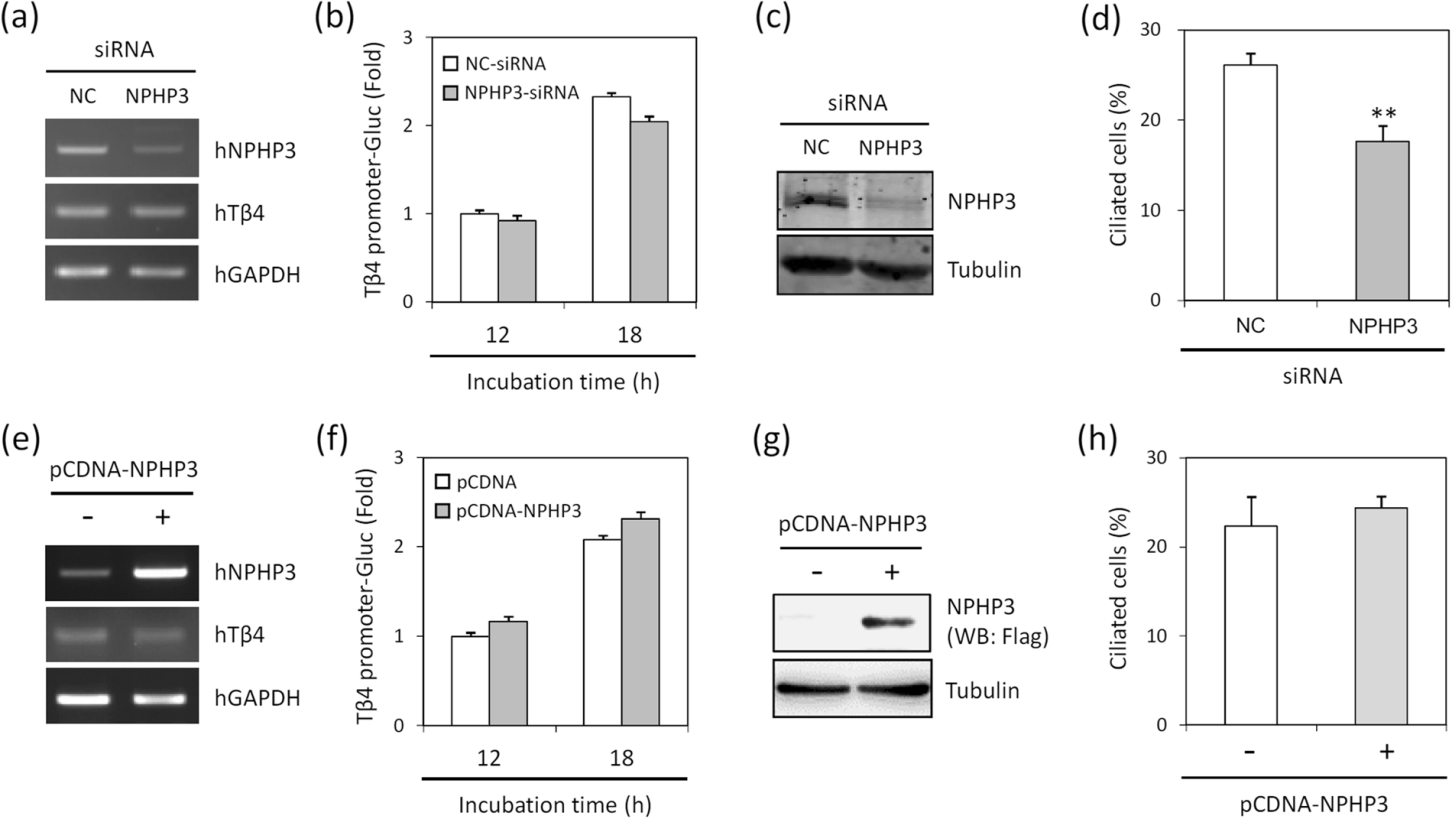
Effect of NPHP3 on Tβ4expression and primary cilia formation. (**a**–**d**) HeLa cells were transfected with AccuTarget™ negative control siRNA (NC) or NPHP3-siRNA for 24 h. (**a**) Expression level of NPHP3 and Tβ4 transcripts were measured by RT-PCR. (**b**) HeLa cells were co-transfected with pEZX-PG02-Tβ4-promoter Gaussia luciferase (Gluc) plasmid. And Gluc activity in cultured media was measured with luminometer using Gluc substrate. Bar graph indicates the mean of Tβ4-promoter activity. (**c**) The protein expression of NPHP3 was detected by western blotting using antibody against NPHP3. (**d**) The cells were followed by the incubation in serum-starved media for 36 h. And the ciliated cells in control cells (white) and NPHP3-knockdown cells (grey) were counted (n > 500 cells). Data in a bar graph represent the means ± SEM. **p < 0.01; significantly different from control cells. (**e**–**h**) HeLa cells were transfected with pCDNA3.1 or pCDNA6-NPHP3 for 24 h. (**e**) Expression level of NPHP3 and Tβ4 transcripts were measured by RT-PCR. (**f**) HeLa cells were co-transfected with pEZX-PG02-Tβ4-promoter Gluc plasmid. Gluc activity in cultured media was measured with luminometer using Gluc substrate. Bar graph indicates the mean of Tβ4-promoter activity. (**g**) The protein expression of NPHP3-flag was detected by western blotting using antibody against flag. (**h**) The cells were followed by the incubation in serum-starved media for 36 h. And the ciliated cells in control cells (white) and NPHP3-overexprssed cells (grey) were counted (n > 500 cells). Processing (such as changing brightness and contrast) is applied equally to controls across the entire image.

We next investigated molecular relevance between Tβ4 and NPHP3. Knockdown of Tβ4 by siRNA-Tβ4 reduced NPHP3 transcripts (Fig. 3a). Also, the decrease of NPHP3-promoter activity in the Tβ4-knockdown cells were confirmed by NPHP3-promoter Gaussia luciferase assay (Fig. 3b). Conversely, overexpression of Tβ4 by transfection of cells with pCMV-Tβ4 increased expression of NPHP3 transcripts (Fig. 3c). The upregulation of NPHP3-promoter activity was consistently detected in the Tβ4-overexpressing cells (Fig. 3d). In contrast, Tβ4 mRNA expression was not influenced by neither knockdown (Fig. 4a) nor overexpression of NPHP3 (Fig. 4e). Tβ4-promoter activity was also not changed by alteration of NPHP3 expression (Fig. 4b,f). These results demonstrate that Tβ4 could be involved in the control of NPHP3 expression.

Next, we tested the effect of NPHP3 on primary cilia formation. The inhibition of NPHP3 protein by siRNA-NPHP3 was confirmed by western blotting (Fig. 4c). The frequency of primary cilia was reduced about 32.4% by the knockdown of NPHP3 (Fig. 4d). Overexpression of NPHP3 slightly increased the percentage of ciliated cells, but this effect was statistically insignificant (Fig. 4h). And the expression of NPHP3-flag was detected using antibody reactive to flag (Fig. 4g). Therefore, these data demonstrate that NPHP3 expression could be essential but its expression only could be insufficient for primary cilia formation. It suggests that Tβ4 could regulate NPHP3 expression leading to modulate primary cilia formation in tumor cells.

### Primary cilia formation is up-regulated by the effect of Tβ4 on upstream of NPHP3-promoter

**Figure 5:**
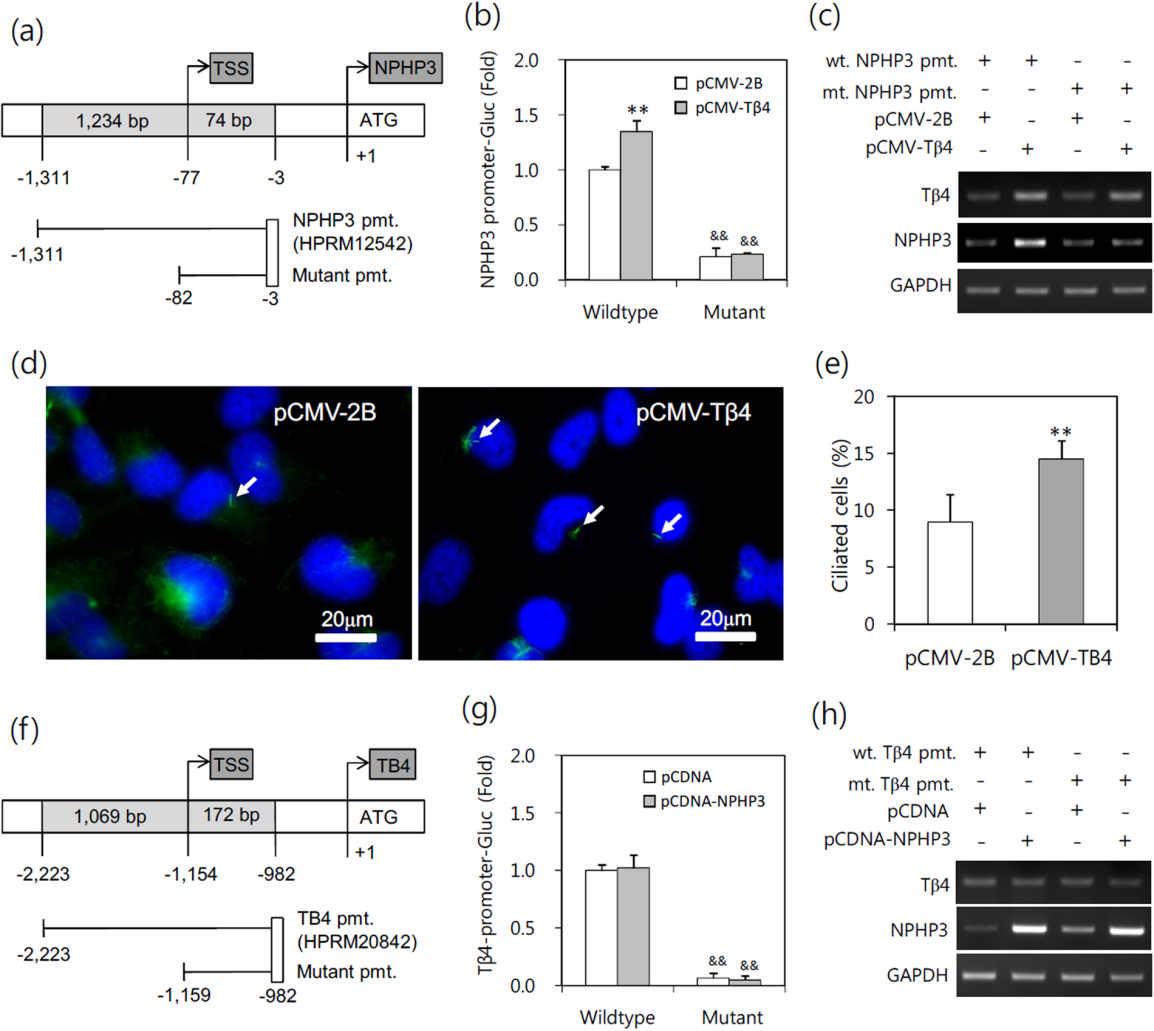
No effect of Tβ4 on upstream-deleted mutant of NPHP3-promoter (pmt.). (**a**) Mutant (mt.) NPHP3-promoter plasmids were prepared from wildtype (wt.) promoter. (**b**–**e**) HeLa cells were transfected with pCMV-2B or pCMV-Tβ4 for 24 h. (**b**) HeLa cells were co-transfected with wildtype or mutant pEZX-PG02-NPHP3-promoter Gluc plasmid. And Gluc activity in cultured media was measured with luminometer using Gluc substrate. Bar graph indicates the mean of NPHP3-promoter activity. (**c**) Expression level of Tβ4 and NPHP3 transcripts were measured by RT-PCR. (**d**) The cells were fixed and stained with antibody against Ac-tubulin (red) and DAPI (blue). (**e**) The ciliated cells in pCMV-2B- (white) or pCMV-Tβ4-transfected group (grey) were counted. (**f**) Mutant (mt.) Tβ4-promoter plasmids were prepared from wildtype (wt.) promoter. (**g,h**) HeLa cells were transfected with pCDNA3.1 or pCDNA6-NPH3 for 24 h. (**g**) HeLa cells were co-transfected with wildtype or mutant pEZX-PG02-Tβ4-promoter Gluc plasmid. And Gluc activity in cultured media was measured with luminometer using Gluc substrate. Bar graph indicates the mean of Tβ4-promoter activity. (**h**) Expression level of Tβ4 and NPHP3 transcripts were measured by RT-PCR. Processing (such as changing brightness and contrast) is applied equally to controls across the entire image (**c,d** and **h**). Data in a bar graph represent the means ± SEM. **p < 0.01; significantly different from control group. ^&&^p < 0.01; significantly different from wildtype promoter plasmid-transfected group.

To confirm the effect of Tβ4 on NPHP3 expression and primary cilia formation, we prepared an upstream-deleted mutant of NPHP3-promoter (Fig. 5a). As shown in Fig. 5b, NPHP3-promoter activity was inhibited by the deletion of upstream (−1,311 ~ −83) in NPHP3-promoter and no changes in mutant NPHP3-promoter activity were detected even in the cells co-transfected with Tβ4. When transcript level of Tβ4 or NPHP3 was detected by RT-PCR, Tβ4 overexpression was observed in groups that were transfected with wildtype or mutant NPHP3-promoter plasmids. NPHP3 mRNA expression was increased by Tβ4 overexpression (Fig. 5c). We also consequently determined the number of cells with primary cilium. Data showed that the frequency of primary cilia was increased about 37.9% by Tβ4 overexpression (Fig. 5d,e). In addition, we prepared an upstream-deleted mutant of Tβ4-promoter (Fig. 5e). As shown in Fig. 5f, Tβ4-promoter activity was inhibited by the deletion of upstream (−2,223 ~ −1,160) in Tβ4-promoter. No changes in wildtype and mutant Tβ4-promoter activity were detected by co-transfection with NPHP3. We observed NPHP3 overexpression in groups that were co-transfected with wildtype or mutant Tβ4-promoter. No changes in Tβ4 mRNA expression levels were detected by NPHP3 overexpression (Fig. 5g). These data re-affirmed that Tβ4 could upregulate NPHP3 expression to influence primary cilia formation. It suggests that Tβ4 might regulate upstream (−1,311 ~ −83) of NPHP3-promoter directly or indirectly through the activation of signaling molecules and any transcription factors.

## Discussion

Several studies have suggested that the assembly and disassembly process of primary cilia is associated with tumorigenesis and tumor malignancy^4–6,8^. Although proteomics research suggested that over 600 proteins are associated in the primary cilia^35^, it still remains unclear how and why those proteins target to the distinct cell compartment. Hence, we would study how ciliary targeting proteins such as NPHP3 are regulated to form primary cilia. The results could be helpful to better understand pathological mechanisms responsible for the cause of ciliopathy-related tumorigenesis.

Tβ4 is an monomer actin-sequestering protein that regulates actin cytoskeleton dynamics^15^. Overexpression of Tβ4is associated with anti-tumor drug resistance^18–20^ and malignancy properties^16,17,21–24^ in various types of cancer cell lines including HeLa cervical cancer cells. In the present study, we performed yeast two-hybrid (Y2H) screening using Tβ4 as a bait and thymus cDNA library as a prey, which led to rescue 10 real positive colonies encoding NPHP3 and one colony encoding 3′ UTR of PMEPA1 that could not be translated into any target protein in NCBI blast (Fig. 2a). NPHP3 is one of the structural component in primary cilia^25,26^, and responsible gene for adolescent nephronophthisis (NPHP)^27^, one of the ciliopathies. Mutation in NPHP3 caused the defect of primary cilia in kidney^28^. In addition, it is such an interest in how a non-translated 3′ UTR of PMEPA1 region cause the activation of the reporter. So, it should be required to clarify the mechanism of action on the activation of the reporter by a non-translated region.

Based on the physical interaction of Tβ4 and NPHP3 in Y2H screening assay, we examined whether Tβ4 expression is involved in primary cilia formation in HeLa cervical cancer cells. The number of ciliated cells was decreased by knockdown of Tβ4 expression (Fig. 1d) and Tβ4 overexpression noticeably increased the number of ciliated cells (Fig. 1f). These findings demonstrate that primary cilia formation could be dependent on Tβ4 expression, even though it is still questionable whether Tβ4 is a ciliary component directly targeting primary cilia formation. When cells were stained with immunofluorescence, no localization of Tβ4 in primary cilia was detected under our experimental conditions.

There are several possibilities to interpret how Tβ4 up-regulate primary cilia formation. Recently, it has been reported that primary cilium elongation was induced by actin depolymerization and the enrichment of many actin-binding proteins inside cilium^36^. Thus, it still remains the possibility that Tβ4 could be translocated into ciliary compartment in any specific manners such as F-actin depolymerization. The second possibility could be a direct effect of Tβ4 to regulate actin cytoskeletal structures on the biogenesis of primary cilia. While stress fibers inhibit cilia formation, cortical actin could provide a scaffold for the cilia formation. Cytochalasin D elongates cilia length and number by disrupting stress fibers^37–39^. Serum starvation disrupted and induced the re-distribution of stress fibers to apical F-actin^40^. Stress fiber is decreased by microinjection of Tβ4^41^ and increased by knockdown of Tβ4^42^. So, it suggests the 3^rd^ possibility that Tβ4 could control ciliogenesis through the regulation of actin stress fibers. Although further studies are necessary to examine those possibilities, Tβ4 could be a novel controller for primary cilia formation.

We also focused on whether ciliary protein, NPHP3, could be regulated by Tβ4 during ciliogenesis, independent of potential effect of Tβ4 on primary cilia formation. We confirmed the direct interaction of NPHP3 and Tβ4 by GST-pulldown assay (Fig. 2b). Tβ4 was co-localized with NPHP3 at the peripheral cell surface instead of within primary cilia (Fig. 2c). The primary cilia formation are rigorously regulated by vesicle trafficking throughout the whole cell^43^. Although ciliary targeting mechanism of NPHP3 has been partially revealed, the accumulation of NPHP3 at basal body is its coiled-coil domain dependent. The myristoylated NPHP3 complex is entered into the ciliary shaft^25,44^ and localized in Inv compartment which is characterized by the accumulation of Inv at the proximal segment of primary cilia and a candidate site for intra-ciliary interaction of Inv, NPHP3 and NEK8^26^. However, it is currently unknown about the trafficking mechanism of NPHP3 from cytosol to the primary cilia. This mechanism could be possibly explained by the principle action of Tβ4 on actin polymerization. Tβ4 interacts with G-actin and supply them to profilin during filament elongation^45^. Then, Tβ4 might be required to locate monomer actins from cytosolic pool to the leading edge^46^. It suggests that NPHP3 could be trafficked by the interaction with Tβ4 to the peripheral compartment from cytosol, which leads to primary cilia formation effectively.

To better understand the correlation between Tβ4 and NPHP3 in ciliary formation, we examined the effect of both gene expression each other. The expression of NPHP3 was dependent on Tβ4 expression (Fig. 3), but not vice versa (Fig. 4a,b,e,f). These data suggest that NPHP3 expression might be necessary to generate primary cilia, but NPHP3 alone is not sufficient to increase them. It explains that the level of NPHP3 expression might be maintained in an appropriate level to keep primary cilia formation physiologically normal. In addition, as Tβ4 modulated primary cilia formation through NPHP3 expression as suggested by the results with NPHP3-siRNA experiment (Fig. 4c,d), it is expected that the increased levels of the protein further increased the number of primary ciliated cells. However, this is not the case even after a massive increase in NPHP3 expression (Fig. 4g,h). In addition, the deletion of upstream (−1,311 ~ −83) in NPHP3-promoter was resulted in the inhibition of NPHP3-promoter activity and no changes in NPHP3 expression even by Tβ4 overexpression (Fig. 5a,b,c,d,e) but not vice versa (Fig. 5f,g,h). Data suggest that Tβ4 might regulate upstream (−1,311 ~ −83) of NPHP3-promoter through direct or indirect activation of signaling molecules and transcription factors. These data also demonstrate that to induce primary cilia formation by modulating NPHP3 expression may require the excessive Tβ4 expression at the same time.

In addition, it could not be ruled out the possibility that Tβ4 might be associated with primary cilia formation independent of NPHP3 as well as NPHP3-dependent pathways. Furthermore, since Tβ4 and NPHP3 are cytoskeleton interactors not to interact directly with DNA, the transactivation of the luciferase reporter could be indirect via other proteins. Therefore, it is required to define specific signaling molecules and transcriptional regulators activated by Tβ4 including HIF-1α or Erk^19,47^, which lead to regulate the expression of Tβ4 and NPHP3.

In conclusion, although many questions remain about the mechanisms underlying Tβ4 action, our results provide a novel evidence to the effect of Tβ4 expression on ciliogenesis through the regulation of NPHP3 in HeLa cervical cancer cells. This work suggests that cooperation of Tβ4 and NPHP3 may be required for primary cilia formation in tumor cells. It also suggests that further investigation would be worthy of the malignancy of ciliated tumor cells regarding Tβ4 action. So, the biological significance of our study is to inform the regulation of ciliogenesis in tumor cells and a novel pathological mechanism on primary cilium-associated diseases.

## Materials and Methods

### Reagents, plasmids and siRNAs

4′,6-diamidno-2-phenylinole (DAPI) was purchased from the Sigma Chemical Co. (St. Louis, MO, USA). Rabbit (AF6796) and mouse (H00007114-B01P) antibodies which are reactive with Tβ4 came from R&D systems Inc (Minneapolis, MN, USA) and Novus Biologicals (Littleton, CO, USA), respectively. Mouse antibodies which are reactive with acetylated tubulin (T7451), and β-tubulin (T4026) were from Sigma-Aldrich Co. (St. Louis, MO, USA). Rabbit antibodies which are reactive with NPHP3 (sc-134745), GFP (sc-138), GST (sc-138) and goat anti-mouse IgG-FITC (sc-2010) were from Santa Cruz Biotechnology, Inc (Santa Cruz, CA, USA). Goat anti-rabbit IgG-Alexa 568 (A-11011), chicken anti-mouse IgG-Alexa 568 (A-21124) and chicken anti-mouse IgG-Alexa 488 (A-21200) were obtained from Invitrogen (Calsbad, CA, USA). Except where indicated, all other materials are obtained from the Sigma Chemical Co. (St. Louis, MO, USA).

pCDNA3.1 and pCMV-2B plasmids were kindly provided by Prof. Young-Joo Jang, College of Dentistry, Dankook University (Cheon-An, Rep. of Korea). pEGFP-C2 plasmid was kindly provided by Prof. Mi-Ock Lee, College of Pharmacy, Seoul National University (Seoul, Rep. of Korea). Flag-tagged pCDNA6-NPHP3 plasmid was were kindly provided by Prof. Carsten Bergmann, Center for Human Genetics, Bioscientia (Ingelheim, Germany). pCMV-Tβ4 and pEGFP-C2-Tβ4 plasmids were generated by customer order for subcloning to Cosmo Genetech Co., Ltd. (Seoul, Rep. of Korea).

Small interference(si) RNAs are customer-ordered to Bioneer (Daejeon, Rep. of Korea). Sequences of siRNAs are as follows: siRNA-Tβ4 with (sense: CCG AUA UGG CUG AGA A; anti-sense: UCG AUC UCA GCC AUA UCG G), siRNA-NPHP3 with (sense: CUG UUG AAA UUC GAC AGA A; anti-sense: UUC UGU CGA AUU UCA ACA G). AccuTarget™ negative control siRNA (SN-1001) was also purchased from Bioneer (Daejeon, Rep. of Korea).

### Cell culture

HeLa human cervical cancer cells (ATCC # CCL-2^TM^) and human embryonic kidney (HEK) 293T cells were obtained from Korea research institute of bioscience and biotechnology (KRIBB) cell bank (Daejeon, Rep. of Korea). Cells were cultured as monolayers in Dullecco’s modified Eagle’s medium (DMEM) with supplement of 10% fetal bovine serum (FBS) (GIBCO, Grand Island, NY, USA), 2 mM L-glutamine, 100 units/ml penicillin and streptomycin (GIBCO, Grand Island, NY, USA). Cells were incubated at 37 °C in a humidified atmosphere of 5% CO_2_ maintenance. For the induction of primary cilia formation, cells were incubated in serum-starved media with 0.1% FBS for 36 h.

### Transfection of nucleic acids

Each plasmid DNA, siRNAs for Tβ4 and NPHP3 and AccuTarget™ negative contol siRNA were transfected into cells as follows. Briefly, each nucleic acid and lipofectamine 2000 (Invitrogen, Calsbad, CA, USA) was diluted in serum-free medium and incubated for 5 min, respectively. The diluted nucleic acid and lipofectamine 2000 reagent was mixed by inverting and incubated for 20 min to form complexes. In the meanwhile, cells were stabilized by the incubation with culture medium without antibiotics and serum for at least 2 h prior to the transfection. Pre-formed complexes were added directly to the cells and cells were incubated for an additional 6 h. Then, culture medium was replaced with antibiotic and 10% FBS-containing DMEM and incubated for 24 h – 72 h prior to each experiment.

NPHP3 was overexpressed by the transfection of cells with pCDNA6-NPHP3 plasmid DNA, which was accompanied with pCDNA3.1 for control group. Tβ4 was overexpressed by the transfection of cells with pCMV-Tβ4 or pEGFP-C2-Tβ4 plasmid DNA,which was accompanied with pCMV-2B or pEGFP-C2 for control group, respectively.

### Detection of primary cilia

For the detection of primary cilia *in vitro*, cells were maintained in serum-starved culture medium for 24–72 h^31–34^. Briefly, HeLa cells were grown on coverslip and then incubated with serum-starved DMEM with 0.1% FBS for 36 h. Cells were fixed with 4% paraformaldehyde for 10 min, washed three times with cold PBS, and permeabilized with PBST (0.1% (v/v) Triton X-100 in PBS) for 10 min. Then, cells were washed three times, and incubated with monoclonal anti-acetylated tubulin antibodies diluted (1:1000) in PBST for 1 h at room temperature. After washing three times with PBS, cells were incubated with FITC-conjugated goat anti-mouse IgG-secondary antibody or goat anti-mouse IgG-Alexa 568 diluted (1:1000) in PBST for 1 h at room temperature. Nucleus was visualized by staining cells with DAPI. After washing with PBS, cells were mounted on glass slide. Primary cilia were observed and photographed at 1,000 x magnification under a fluorescence microscope (Nikon, Tokyo, Japan).

### Immunofluorescence staining

Cells with the indicated condition were grown on coverslip for 24 h and fixed with 4% paraformaldehyde (PFA) solution freshly prepared in phosphate buffered saline(PBS) for 10 min. Then, cells were permeabilized with 0.1% Triton X-100 in PBS and stained with antibodies to hTβ4 or hNPHP3. Secondary antibodies used were as follows: goat anti-mouse IgG-FITC or chicken anti-mouse IgG-Alexa 488 for hTβ4 and goat anti-rabbit IgG-Alexa 568 for NPHP3. Nucleus was visualized by staining cells with DAPI. Cells were observed and photographed at 1,000 x magnification under a fluorescence microscope (Nikon, Tokyo, Japan).

### Gaussia luciferase assay for promoter activity

Pre-designed promoters for NPHP3 (NM_153240) and Tβ4 (NM_021109) were obtained from GeneCopoeia Inc. (Rockville, MD, USA). NPHP3-promoter (HPRM12542) was 1,309 bp (−1,311 ~ −3) upstream from starting codon for NPHP3 transcription in Homo sapiens 3 BAC RP11-39E4 (AC055732.16). Tβ4-promoter (HPRM20842) was 1,242 bp (−2,223 ~ −982) upstream from starting codon for Tβ4 transcription in Homo sapiens X BAC RP11-102M2 (AC139705.4). Schematic figures and sequences of promoters were shown in Supplementary Fig. S3.

Wildtype and mutant promoters were cloned into Gaussia luciferase (Gluc) reporter plasmid vector, pEZX-PG02. NPHP3 mutant promoter 80 bp (−82 ~ −3) was prepared by the deletion of wildtype upstream (−1,311 ~ −83). Tβ4 mutant promoter 178 bp (−1,158 ~ −982) was prepared by the deletion of wildtype upstream (−2,223 ~ −1,159). HeLa cells were transfected with the NPHP3-Gluc or Tβ4-Gluc plasmids using lipofectamine 2000 (Invitrogen, Carlsbad, CA, USA) as described above. Then, cells were incubated for an appropriate time. Secreted Gluc reporter protein was obtained by the collection of culture-conditioned media after the indicated time intervals. Gluc activity of reporter protein was measured by BioLux^®^ Gluc assay kit (New England BioLabs, Ipswich, MA, USA) including coelenterazine as a substrate for Gluc according to the manufacturer’s protocol. Luminescence was detected by using Lumet 3, LB 9508 tube luminometer (Berthold Technologies GmbH & Co. KG, Bad Wildbad, Germany).

### Reverse transcription polymerase chain reaction (RT-PCR)

Total RNA was extracted by using TRizol reagent (Invitrogen, Calsbad, CA, USA). Complementary DNA (cDNA) was synthesized from 1 μg of isolated total RNA, oligo-dT_18_, and superscript reverse transcriptase (Bioneer, Daejeon, Rep. of Korea) in a final volume of 20ul. For standard PCR, 1 μl of template cDNA was amplified with Taq DNA polymerase. PCR amplification was performed with 25~35 thermocycles for 30 sec at 95 °C, 30 sec at 55 °C, and 60 sec at 72 °C using human (h) oligonucleotide primers specific for hTβ4 (sense: ACA AAC CCG ATA TGG CTG AG; anti-sense: CCT CCA AGG AAG AGA CTG AA), hNPHP3 (sense: AGC GAA ATA CCA AGC AAT GG; anti-sense: TGG AAG GTT CAC TTC CCA AG), hGAPDH (sense: GAA GGT GAA GGT CGG AGT C; anti-sense: GAA GAT GGT GAT GGG ATT TC). Amplified PCR products were separated by 1.0~1.5% agarose gel electrophoresis and detected on Ugenius 3^®^ gel documentation system (Syngene, Cambridge, United Kingdom).

### Western blotting

Cells were lysed in ice-cold RIPA buffer (Triton X-100,) containing protease inhibitor (2 μg/ml aprotinin, 1 μM pepstatin, 1 μg/ml leupeptin, 1 mM phenylmetylsufonyl fluoride (PMSF), 5 mM sodium fluoride (NaF) and 1 mM sodium orthovanadate (Na_3_VO_4_)). The protein concentration of the sample was measured by using SMART^TM^ BCA protein assay kit (Pierce 23228) from iNtRON Biotech. Inc. (Seoul, Rep. of Korea). Same amount of heat-denatured protein in sodium dodecyl sulfate (SDS) sample buffer was separated in sodium dodecyl sulfate polyacrylamide gel electrophoresis (SDS-PAGE), and then transferred to nitrocellulose membrane by using electro-blotter. Equal amount of loaded sample on membrane was verified by ponceau S staining. The membrane was incubated with blocking solution (5% non-fat skim milk in Tris-buffered saline with Tween 20 (TBST)), and then followed by incubation with the specific primary antibodies. Horse radish peroxidase (HRP)-conjugated secondary antibody was used for target-specific primary antibody. Target bands were visualized by the reaction with enhanced chemi-luminescence (ECL) (Dong in LS, ECL-PS250). Immuno-reactive target bands were detected by X-ray film (Agfa healthCare, CP-BU new) or chemiluminescence imaging system Fusion Solo (VilberLourmat Deutschland GmbH, Germany).

### Yeast two-hybrid screening assay

Yeast two-hybrid screening was performed by Panbionet Corp. (http://panbionet.com, Pohang, Rep. of Korea). Briefly, yeast two-hybrid assay was performed by using was performed by using GAL4 DNA-binding domain (BD)-fused Tβ4 as a bait and hybrid library of the human thymus cDNA activation domain (AD) as prey. Tβ4 bait, TMSB4X gene, was cloned into EcoR I/BamH I sites of pGBKT vector containing DNA binding domain of GAL4 (GAL4-BD). The cDNA inserts in library were cloned as Eco RI/Xho I fragments in pACT2 containing GAL4 activation domain (GAL4-AD). The PBN204 yeast strain was co-transformed with the bait DNA and prey library vectors. The PBN204 contains three reporter genes, *URA3*, *ADE2* and *lacZ* that are under the control of each different GAL4-binding promoter. For the first screening, the transformants were spread on selection medium in the absence of leucine, tryptophan, and uracil (SD-LWU). After selection of yeast colonies on uracil-deficient media, the uracil-positive colonies were spread on adenosine-deficient medium (SD-LWA) or tested for beta-galactosidase activity. In order to confirm the interaction, prey parts of DNAs from candidates which satisfied three reporter genes expression, were amplified by PCR and then reintroduced into yeast PBN204 strain with Tβ4 bait or negative control plasmid. Colonies were confirmed in SD-LWU and SD-LWA medium. Then, DNA sequencing and restriction enzyme digestion were performed to select the real positive colonies out of colonies grown in SD-LWU and SD-LWA. Prey’s gene in each positive colony was identified by running alignment of DNA sequence in NCBI blast.

### GST-pulldown assay

Intracellular interaction of Tβ4 and NPHP3 was determined by GST-pulldown assay. In brief, HEK293T cells were transfected with pSG5-Tβ4 and pCDNA6-NPHP3 plasmid DNA. The cells were lysed in 0.5% NP-40 lysis buffer (20 mM Tris-HCL at pH 8.2, 150 mM NcCl) containing protease inhibitors (2 μg/ml aprotinin, 1 μM pepstatin, 1 μg/ml leupeptin, 1 mM phenylmetylsufonyl fluoride, 5 mM sodium fluoride and 1 mM sodium orthovanadate). Then, 500 μg proteins of cell lysates were incubated with glutathione agarose 4B bead (Incospharm, 1101-1) with continuous rotation at 4 °C for 1 h. Washing step with PBS was followed to remove non-specific bound proteins to the bead for 5 times. And then, proteins bound in the agarose beads were eluted in sodium dodecyl sulfate (SDS) sample buffer with boiling and analysed by running on SDS-PAGE. Immunoblot analysis using specific antibodies which are reactive with GST or flag was subjected for the detection of Tβ4-GST or NPHP3-flag, respectively.

### Statistical analysis

Experimental differences were verified for statistical significance using ANOVA and student’s t-test. P value of < 0.01 was considered to be significant.

## Supplementary information

Dataset 1, 2, 3
