# Supplementary material for: Thymosin β-4 is a novel regulator for primary cilium formation by nephronophthisis 3 in HeLa human cervical cancer cells
Source: Sci Rep. 2019 May 2;9:6849. doi: 10.1038/s41598-019-43235-1 (PMC6497666; doi:10.1038/s41598-019-43235-1)
Supplement: Supplementary file 1 — Dataset 1, 2, 3 [file 41598_2019_43235_MOESM1_ESM.doc]

**Thymosin β-4 is a novel regulator for primary cilium formation by nephronophthisis 3 in HeLa human cervical cancer cells**

Jae-Wook Lee^1^, Hong Sug Kim^2^ & Eun-Yi Moon^1^, *

^1^Department of Bioscience and Biotechnology, Sejong University, Seoul 05006, Republic of Korea

^2^Macrogen Inc., 254, Beotkkot-ro, Geumcheon-gu, Seoul 08511, Republic of Korea

* Corresponding author: eunyimoon@sejong.ac.kr


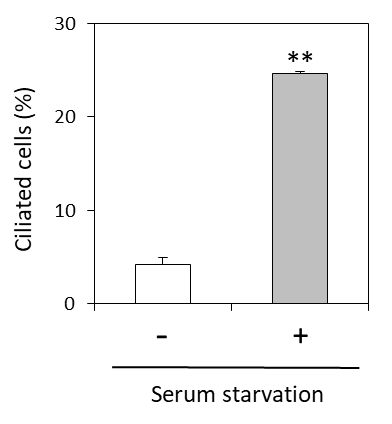


**Supplementary Figure S1.** HeLa cells were ciliated under serum stave condition. HeLa cells were incubated in serum-containing media (5% FBS) or serum-starve media (0.1%) for 36h. Cells were fixed and stained with antibody against acetylated tubulin. The representative mean percentage of ciliated cells were presented in bat graph. Data in a bar graph represents the means ± SEM. **p<0.01; significantly different from serum-containing control cells.

**Supplementary Figure S2.** DNA sequence for each prey. (a-d) Positive yeast colonies 1 ~ 11 were obtained from yeast two-hybrid screening assay. The cDNA inserts in positive clones were cloned as Eco RI/Xho I fragments in pACT2 containing GAL4 activation domain (GAL4-AD). Prey DNA from each clone was amplified by its transformation into *Escherichia coli* and purified after the incubation. Then, DNA in each positive clone was sequenced to identify prey gene by running alignment of DNA sequence in NCBI blast. ^Ñ^GAATTC underlined indicated the EcoRI site for insertion of prey gene. Grey characters indicated nucleotides that were aligned to NPHP3 (a-c). The activation domain (AD) in prey #1 (69^th^ aa ~ 223^rd^ aa) was fused in frame to ‘C’ of **CTG** which encoded the 69^th^ aa of nephronophthisis 3 **(**NPHP3). ^♦^**C** was the 259^th^ nucleotide of NPHP3 mRNA (a). AD in clones prey #2 ~ #10 (89^th^ aa ~ 223^rd^ aa) was fused in frame to ‘C’ of **GCC** which encoded the 89^th^ aa of NPHP3. ^♦^**C** was the 319^th^ nucleotide of NPHP3 mRNA (b). AD was fused to 3’ UTR of prostate transmembrane protein, androgen induced 1 (PMEPA1), transcript variant 1. ^♦^**C** was 1272^nd^ nucleotide of PMEPA1 mRNA (c). Insertion of prey was confirmed by reverse sequencing of DNA from prey #7 as the representative for all preys. CTCGAG indicated the XhoI site for insertion of prey gene.

**Supplementary Figure S3.** Schematic figures and sequences of pre-designed NPHP3 (NM_153240) and Tβ4 (NM_021109) promoters. (a) NPHP3 promoter (HPRM12542) was 1,309 bp (-1,311 ~ -3) upstream from starting codon, ATG, of coding sequence (CDS) for NPHP3 transcription. NPHP3 promoter covers 1,234 bp upstream and 74 bp downstream from transcription starting site (TSS). (b) TSS in sequences of NPHP3 promoter was shown with bold capital letter underlined. (c) Tβ4 promoter (HPRM20842) was 1,242 bp (-2,223 ~ **-**982) upstream from starting codon of CDS for Tβ4 transcription. Tβ4 promoter covers 1,069 bp upstream and 172 bp downstream from TSS. (d) TSS in sequences of Tβ4 promoter was shown with bold capital letter underlined.
